# Carotenoid Biosynthetic and Catabolic Pathways: Gene Expression and Carotenoid Content in Grains of Maize Landraces

**DOI:** 10.3390/nu6020546

**Published:** 2014-01-28

**Authors:** Rafael da Silva Messias, Vanessa Galli, Sérgio Delmar dos Anjos e Silva, Cesar Valmor Rombaldi

**Affiliations:** 1 Embrapa Temperate Agriculture, BR 396, Km 78, P.O. Box 403, Pelotas-RS 96010-900, Brazil; E-Mails: vane.galli@yahoo.com.br (V.G.); sergio.anjos@embrapa.br (S.D.A.S.); 2 Federal University of Rio Grande do Sul, Biotechnology Center, Av. Bento Gonçalves 9500, P.O. Box 15005, Porto Alegre-RS 91501-970, Brazil; 3 Federal University of Pelotas, Eliseu Maciel Agronomy College, Campus Universitário s/n, P.O. Box 354, Pelotas-RS 96010-900, Brazil; E-Mail: cesarvrf@ufpel.edu.br

**Keywords:** *Zea mays* L., landrace varieties, carotenoids, carotenoid-related genes, carotenoid cleavage dioxygenases

## Abstract

Plant carotenoids have been implicated in preventing several age-related diseases, and they also provide vitamin A precursors; therefore, increasing the content of carotenoids in maize grains is of great interest. It is not well understood, however, how the carotenoid biosynthetic pathway is regulated. Fortunately, the maize germplasm exhibits a high degree of genetic diversity that can be exploited for this purpose. Here, the accumulation of carotenoids and the expression of genes from carotenoid metabolic and catabolic pathways were investigated in several maize landraces. The carotenoid content in grains varied from 10.03, in the white variety MC5, to 61.50 μg·g^−1^, in the yellow-to-orange variety MC3, and the major carotenoids detected were lutein and zeaxanthin. *PSY1* (phythoene synthase) expression showed a positive correlation with the total carotenoid content. Additionally, the *PSY1* and *HYD3* (ferredoxin-dependent di-iron monooxygenase) expression levels were positively correlated with β-cryptoxanthin and zeaxanthin, while *CYP97C* (cytochrome P450-type monooxygenase) expression did not correlate with any of the carotenoids. In contrast, *ZmCCD1* (carotenoid dioxygenase) was more highly expressed at the beginning of grain development, as well as in the white variety, and its expression was inversely correlated with the accumulation of several carotenoids, suggesting that CCD1 is also an important enzyme to be considered when attempting to improve the carotenoid content in maize. The MC27 and MC1 varieties showed the highest *HYD3*/*CYP97C* ratios, suggesting that they are promising candidates for increasing the zeaxanthin content; in contrast, MC14 and MC7 showed low *HYD3*/*CYP97C*, suggesting that they may be useful in biofortification efforts aimed at promoting the accumulation of provitamin A. The results of this study demonstrate the use of maize germplasm to provide insight into the regulation of genes involved in the carotenoid pathway, which would thus better enable us to select promising varieties for biofortification efforts.

## 1. Introduction

The grass family (Poaceae), including maize, wheat, rice, sorghum, and millet, contains the major staple crops. Maize is one of the most important cereal crops worldwide, as evidenced by the multiple ways in which it is exploited. Maize provides food and feed and is a resource for many unique industrial and commercial products, ranging from textiles and oil to pharmaceutical products. Maize alone is responsible for providing 15% of the total protein and 20% of the total calories in the human diet [1]. In addition to its nutritional importance, maize represents one of the most important sources of carotenoids [2,3].

Carotenoids are a complex class of isoprenoid pigments, which provide nutritional and functional value as both provitamin A and non-provitamin A compounds, such as lutein and zeaxanthin. In humans, these carotenoids have been implicated in preventing various eye and cardiovascular diseases, as well as several types of age-related diseases, most likely via their role as antioxidants and/or as regulators of the immune system [4]. In this context, maize grain has been considered an important candidate in metabolic engineering approaches aimed at specifically increasing the levels of zeaxanthin, lutein and provitamin A carotenoids in crop species, to alleviate vitamin A deficiency, which predisposes an estimated 250 million or more children worldwide to visual impairment and blindness [5].

**Figure 1:**
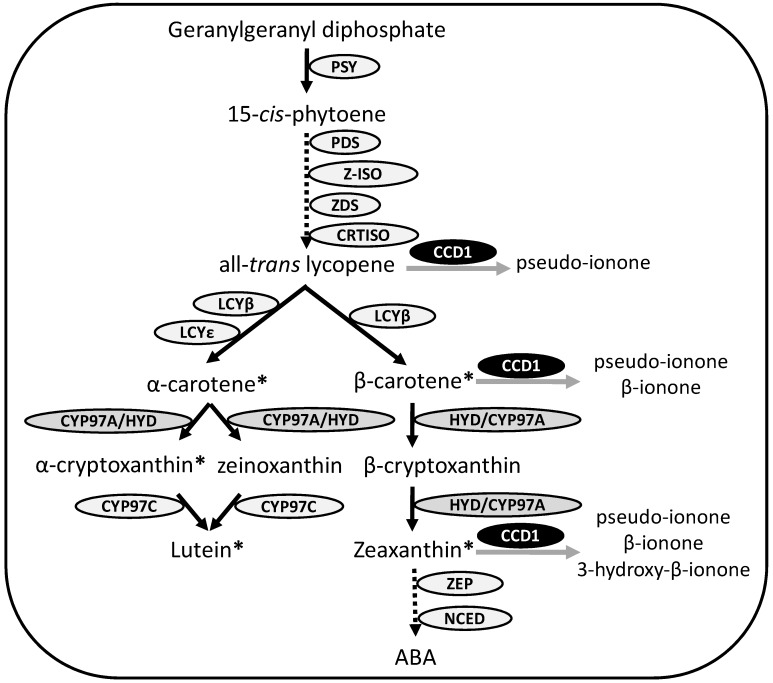
Simplified carotenoid biosynthetic pathway. Enzymatic reactions are represented by arrows. Dotted lines indicates that there are steps not shown. PSY, phytoene synthase; PDS, phytoene desaturase; ZDS, ζ-carotene desaturase; CRTISO, carotene isomerase; Z-ISO, 15-*cis*-ζ-carotene isomerase; LCYβ, lycopene beta cyclase; LCYε, lycopene epsilon cyclase; CYP97A, cytochrome P450-type β-hydroxylase; HYD, ferredoxin-dependent di-iron monooxygenase; CYP97C, cytochrome P450-type monooxygenase; ZEP, zeaxanthin epoxidase; NCED, 9-*cis*-epoxycarotenoid dioxygenase; ABA, abscisic acid; CCD1, carotenoid dioxygenase. CCD1 in dark circles indicates the action of CCD1 in the metabolic pathway, according to Sun *et al.* [9]. Asterisks (*) represent compounds that showed a negative correlation with *ZmCCD1* expression in the present study.

Most efforts to alleviate vitamin A deficiency have focused on the manipulation of carotenoid biosynthetic pathway enzymes to improve the nutritional content or functional properties of maize grains [6]. In plants, the simplified carotenoid biosynthetic pathway begins with the formation of phytoene from geranylgeranyl pyrophosphate, a step mediated by phytoene synthase (PSY) [4,7]. Phytoene is then converted to all-translycopene by four desaturation and two isomerization reactions [8]. Lycopene is cyclized to give rise to two branches in the carotenoid biosynthetic pathway, the β,ε branch and the β,β branch. The generation of α-carotene from the β,ε branch is dependent on lycopene epsilon cyclase (LCYε) and lycopene beta cyclase (LCYβ); the generation of β-carotene from the β,β branch is dependent on LCYβ. Hydroxylation of the two β-ionone rings in β-carotene, the most potent dietary source of vitamin A, leads to the formation of zeaxanthin via β-cryptoxanthin, while hydroxylation of one β-ring and one ε-ring in α-carotene (and therefore half the provitamin A potential compared to β-carotene) gives rise to lutein via zeinoxanthin or α-cryptoxanthin. Hydroxylation of the ε-ring of α-carotene is mediated by ε-hydroxylase CYP97C (cytochrome P450-type monooxygenase), and hydroxylation of the β-rings of α and β-carotene are mediated by CYP97A (cytochrome P450-type β-hydroxylase) and/or HYD (ferredoxin-dependent di-iron monooxygenase) (Figure 1).

The yellow pigments of maize grains are also affected by the oxidative cleavage performed by a family of carotenoid cleavage dioxygenases (CCDs) and 9-*cis*-epoxycarotenoid dioxygenases (NCEDs) (Figure 1), which catalyze the cleavage of carotenoids at specific double bonds resulting in the production of apocarotenoids [10,11]. These compounds play roles in a variety of biological processes [12]. For example, abscisic acid (ABA) is involved in regulating plant responses to various environmental stresses, particularly drought and salinity stress, and in long-distance signaling within the plant [13]. Other apocarotenoids contribute to the flavor and/or aroma of flowers and/or fruits of several agricultural species [10]. Currently, the only CCD that has been characterized in detail in maize is CCD1. Arabidopsis *CCD1* mutants have increased seed carotenoid content [14], and when a short version of the recombinant *ZmCCD1* was expressed in *E. coli* cells producing lycopene, β-carotene and zeaxanthin, colonies failed to develop the yellow-to-orange color [9]. These results imply that the cleavage promoted by *Zm*CCD1 may reduce the availability of provitamin A carotenoids in the grains.

Therefore, the success of efforts toward biofortification with provitamin A and/or non-provitamin A compounds in maize grains is clearly dependent on the understanding of the mechanisms involved in the regulation of the two branches of the carotenoid biosynthetic pathway, as well as on the regulation of the catabolic pathway [15]. Preliminary studies have demonstrated the relationship between carotenoid levels and several carotenogenic genes [16,17]. Using genetic transformation, several gene functions have been elucidated, and several new products, such as Golden Rice (*Oryza sativa*) have been developed [18,19,20,21,22,23]. However, to the present, there is no report regarding the expression profile of *ZmCCD1* during maize grain development, and the correlation between the expression of *ZmCCD1* and carotenoid content in a large set of maize varieties has yet to be examined. In addition, the bottlenecks involved in the regulation of the two branches of carotenoid biosynthetic pathway are not fully understood. Although the enzymes involved in the hydroxylation of β-rings of α- and β-carotene in maize have been identified, a knowledge gap exists with regard to the contributions of CYP97A and HYD in each branch of the carotenoid pathway; it remains unknown whether both enzymes potentially act in both branches or each enzyme has a preferred substrate.

Maize inbred lines with natural genetic variation in the content of carotenoids may be exploited to provide information about the regulation of this metabolic pathway. The study of these inbred lines also could help to identify the most suitable materials to use in conventional breeding or transgenic research to improve carotenoid levels in maize grains and/or to increase plant resistance to biotic and abiotic stresses [2,19,24]. In the present study, the transcriptional patterns of key genes affecting grain carotenoid accumulation were evaluated in samples from multiple maize grain developmental stages and from several maize landraces, all different in terms of carotenoid content and diversity. As such, it was possible to gain new insight into the regulation of the carotenoid biosynthetic and cleavage pathways, thus improving our ability to select promising varieties for breeding programs.

## 2. Experimental Section

### 2.1. Experimental Design and Varieties

Twenty-two landrace varieties, with large variations in grain color, hardness and shape, and the commercial hybrid maize variety 30F53 (Pioneer^®^) were grown in a field in triplicate, in 10 m plots with four rows. Cobs were covered at the beginning of their formation and were manually pollinated to avoid cross-pollination.

### 2.2. Sample Collection and Storage

Five cobs of each biological replicate (*n* = 3) were collected from the central lines of the block at 22 days after pollination (DAP) and at 48 DAP (grains at harvest—approximately 20% moisture). Grain samples from the commercial maize hybrid were collected at 0, 10, 13, 16, 19, 21 and 25 DAP to evaluate gene expression profiles during grain development. The grains from each biological replicate were pooled and homogenized, then immediately frozen in liquid nitrogen and stored at −80 °C until analysis.

### 2.3. Quantification of Carotenoids

Lyophilized grains collected at 48 DAP were used to quantify the content of carotenoids. The total carotenoid content was determined using a method adapted from Rodriguez-Amaya and Kimura [25] and calculated based on a β-carotene curve. Grains had been previously subjected to hot hydration at 85 °C for 15 min to facilitate carotenoid extraction. The levels of α-carotene, β-carotene, α-cryptoxanthin, β-cryptoxanthin, lutein and zeaxanthin in the grains were determined using the method described by Kimura *et al.* [26].

### 2.4. Primer Design and Gene Expression Analysis

To evaluate gene expression, whole maize grains were crushed with a mortar and pestle using liquid nitrogen. Three biological replicates of each sample were subjected to total RNA isolation using a modified CTAB (hexadecyltrimethylammonium bromide) protocol, as previously described [27]. The RNA quality was evaluated by visualization on a 1% agarose gel after electrophoresis and by spectrometry using the A_260_/A_280_ and A_260_/A_230_ ratios. The RNA concentration was measured in a Qubit^®^ fluorometer (Invitrogen, São Paulo, Brazil). Total RNA (1 μg) was digested with 1 U DNase I and reverse transcribed using the enzyme M-MLV, according to the manufacturer’s instructions (Invitrogen). Specific primers for the amplification of maize genes associated with carotenoid metabolism (*PSY1*, *HYD3*, *CYP97C* and *ZmCCD1*) and the reference genes encoding tubulin (*TUB*), ubiquitin (*UBI*) and actin (*ACT*) were designed based on the sequences extracted from GenBank (National Center for Biotechnology Information, U.S. National Library of Medicine, Bethesda, MD, USA) using Vector NTI10 software (Invitrogen) (Table 1). All of the amplicons were designed to be less than 150 bp in length.

The cDNAs were amplified by RT-qPCR in a final volume of 20 μL containing 1 μL cDNA, 10 μL Platinum^®^ SYBR Green qPCR Supermix-UDG (Invitrogen), and 2–5 pmol of each primer. Amplification was standardized in a 7500 Fast Real-Time Thermal Cycler (Applied Biosystems, São Paulo, Brazil) using the following conditions: 50 °C for 20 s, 95 °C for 10 min followed by 45 cycles of 95 °C for 15 s and 60 °C for 60 s. The PCR products for each primer set were subjected to melting curve analysis to verify the presence of primer dimers or nonspecific amplicons. The melting curve analysis ranged from 60 to 95 °C, with the temperature increasing stepwise by 1%. The results were calculated using the 2^−ΔΔ*C*T^ method [28].

**Table 1:** Primer sequences and amplicon characteristics of evaluated genes.

Gene	GenBank Accession	Forward Primer Sequence	Reverse Primer Sequence	Amplicon Length	Product TM °C ^a^
*TUB*	NM_001111988.1	AGAACTGCGACTGCCTCCAAAGG	AGATGAGCAGGGTGCCCATTC	84	82.6
*ACT*	J01238.1	CATGGAGAACTGGCATCACACCTT	CTGCGTCATTTTCTCTCTGTTGGC	118	84.3
*UBI*	M73785.1	GTTTAAGCTGCCGATGTGCCTG	GACACGACTCATGACACGAACA	89	82.3
*PSY1*	FJ971251.1	GACAGATGAGCTTGTAGATGGGC	TCAGAGAGAGCGGCATCAAGCA	129	83.1
*HYD3*	AY844958	GGGGATTACGCTGTTCGG	GTGGTGTATCTTGTGCGAGG	130	89.5
*CYP97C*	BE552887	GTTGACATTGGATGTGATTGG	AACCAACCTTCCAGTATGGC	150	78.7
*CCD1*	DQ100346.1	CTGCTGTGGATTTTCCTCGTG	TATGATGCCAGTCACCTTCGC	104	82.3

^a^ The melting temperature was calculated by SDS version 1.1 software in a 7500 Fast Real-Time Thermal Cycler (Applied Biosystems, São Paulo, Brazil).

### 2.5. Statistical Analysis

Pearson’s test (*p* ≤ 0.01) was used and the analyses were conducted using GraphPad Prism 4 (GraphPad Software Inc., San Diego, CA, USA).

## 3. Results and Discussion

### 3.1. Time-Series Expression Profiles of Carotenoid-Related Genes during Multiple Grain Developmental Stages of the Hybrid 30F53

**Figure 2:**
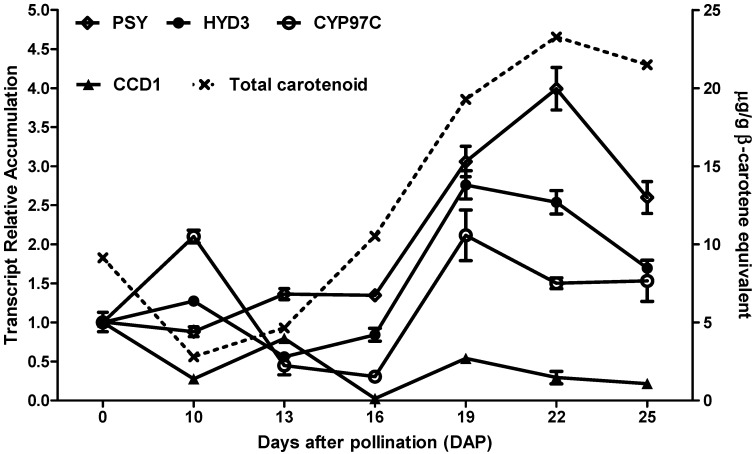
Time-series expression of carotenoid-related genes and total carotenoid content of the commercial 30F53 hybrid maize grain at 0, 10, 13, 16, 19, 22 and 25 days after pollination (DAP). Means of three replicates ± SD are shown. Day 0 was used as a reference sample for gene expression analysis, calculated using the 2^−ΔΔ*C*T^ method.

Knowledge of the expression profile of genes involved in the carotenoid metabolic and catabolic pathways during grain development is important, as it can provide information about the regulation of these pathways over time, when correlated with carotenoid accumulation. Therefore, the expression profiles of several carotenogenic genes were assessed during maize grain development and compared to the total carotenoid content in the hybrid 30F53. Among the carotenogenic genes, we evaluated the expression of *PSY1* because it encodes the first enzyme in the carotenoid biosynthetic pathway, as well as the expression of *HYD3* and *CYP97C* because they encode the last enzymes in the same pathway; *HYD3* is the last enzyme in the β,β branch, and *CYP97C* is the last enzyme in the β,ε branch. Therefore, we aimed to obtain information regarding not only the overall flux of the carotenoid pathway but also the regulation of the flux direction to each branch from the pathway. Using this approach, we observed that the expression level of *PSY1* was relatively low until 16 DAP and then increased sharply from 16 DAP to 22 DAP, reaching the peak of expression during this time before declining (Figure 2). *HYD3* and *CYP97C* behaved similarly, showing increased expression levels at 10 DAP, decreasing sharply until 13/16 DAP, and then increasing again, with their expression peaks occurring at 19 DAP. Overall, the carotenogenic genes analyzed showed high expression levels during the later stages of maize grain formation (between 19 and 22 DAP), during which the presence of carotenoids is necessary to protect the grain against oxidative damage caused by UV radiation. Herein, *PSY1* and *HYD3* expression showed correlations with total carotenoid levels of approximately 92% and 81%, respectively (*p* < 0.05). Moreover, *HYD3* expression showed 87% and 73% correlation with *PSY1* and *CYP97C* expression (*p* < 0.05), respectively, indicating the strong relationship between these genes acting in the same metabolic pathway. The correlation between *PSY1* expression and total carotenoids in hybrid maize grains has been previously reported [17,29]. This correlation is thought to be a result of the *PSY* genes having been under strong selection.

The role of CCDs in the degradation of maize carotenoids remains unknown; therefore, the expression profile of *ZmCCD1*, the only well-characterized CCD in maize, was evaluated during maize grain development. In this study, *ZmCCD1* was more highly expressed at the beginning of grain formation (at 0 and 13 DAP), and expression declined after 13 DAP (Figure 2). These results suggest that *CCD1* expression is induced at the start of pigment production and is down regulated when biosynthesis is accomplished. A similar expression pattern of *CCD1* was observed during seed development in the coffee grain (*C. canephora*) [30], as well as during seed maturation in *Bixa orellana* [31]. The importance of this gene in the depletion of the carotenoid pool during grain maturation has been demonstrated in the Arabidopsis *CCD1* knockout mutant, which had a normal appearance but high levels of mature grain carotenoids [14]. Additionally, a maize inbred line with a high *ZmCCD1* copy number exhibited a low endosperm carotenoid content [32]. Therefore, CCD1-associated carotenoid catabolism likely has negative consequences in terms of the nutritional quality of the grain. In the present study, *ZmCCD1* expression was negatively correlated (*r* = −0.53, *p* < 0.01) with total carotenoid content, suggesting that the conversion of carotenoids to apocarotenoids represents an important step that will need to be considered when attempting to improve the carotenoid content in maize grains. Here, we have also provided temporal information on gene expression that will guide future metabolic engineering efforts. It is important to consider, however, that it may not be possible to extrapolate the expression profiles observed during the development of grain from the 30F53 variety to all maize varieties.

### 3.2. Carotenoid Profile and the Expression Pattern of Carotenoid-Related Genes in Maize Landraces

**Figure 3:**
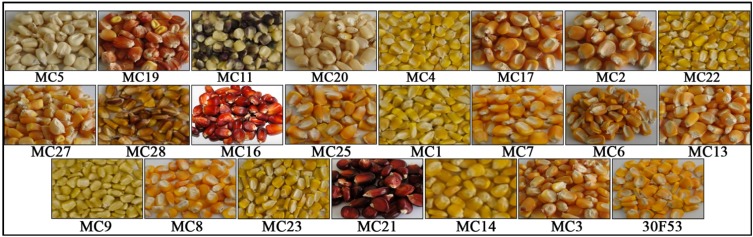
Grains from maize landrace varieties used in the study. The commercial hybrid 30F53 is also included here. The white maize grain (MC5) was used as a control variety.

To obtain information about the regulatory mechanisms involved in carotenoid biosynthesis, the expression levels of genes involved in the metabolic and catabolic pathways of carotenoids were evaluated in different maize landraces (Figure 3) and compared with the levels of specific carotenoids in mature kernels.

**Figure 4:**
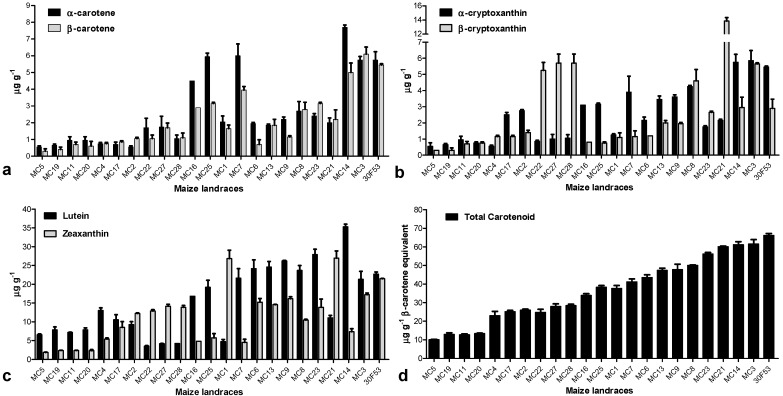
Carotenoid contents in mature grains (48 DAP) from maize varieties. (**a**) Alpha-carotene and β-carotene; (**b**) α-cryptoxanthin and β-cryptoxanthin; (**c**) lutein and zeaxanthin; and (**d**) total carotenoids. The means of three replicates ± SD are shown. Inbred lines are as shown in Figure 3.

The evaluated maize landraces exhibited diverse phenotypes, with colors varying from white, light and dark yellow, orange, red and purple (Figure 3), and the grains likely contained a large range of carotenoid contents and high diversity (Figure 4), features that are useful for investigating pathway regulation. The total carotenoid content in maize grains at harvest varied from 10.03 (in the white variety MC5) to 61.50 μg·g^−1^ (in the yellow-to-orange variety MC3), with the hybrid variety having 66.15 μg·g^−1^ (Figure 4). The most abundant carotenoids were lutein (ranging from 3.50 to 35.30 μg·g^−1^) and zeaxanthin (1.85 to 26.95 μg·g^−1^). The varieties studied here also contained 0.55 to 7.70 μg·g^−1^ of α-carotene, 0.30 to 6.10 μg·g^−1^ of β-carotene, 0.55 to 5.85 μg·g^−1^ of α-cryptoxanthin, and 0.30 to 13.85 μg·g^−1^ of β-cryptoxanthin. As expected, the white variety (MC5) had the lowest levels of all evaluated carotenoids, except lutein. The MC14 variety had the highest levels of carotenoids from the β,ε-branch of the carotenoid pathway (α-carotene, α-cryptoxanthin and lutein). In contrast, there was no single variety that had the highest levels of all evaluated carotenoids from the β,β-branch (Figure 4). A positive significant correlation was also observed between the total carotenoid content and the content of each of the evaluated individual carotenoids. For example, there was a correlation of 79% (*p* < 0.01) between β-carotene and the total carotenoid content (Table 2).

**Table 2:** Correlation of mRNA levels with carotenoid content in maize grains. Pearson correlation for the comparison of transcript levels in grains at 22 days after pollination (DAP) and kernel carotenoid content in mature grains (48 DAP) from the maize varieties was performed using GraphPad Prism 4 (GraphPad Software Inc., San Diego, CA, USA) to test the statistical significance of the relationship. * indicates *p* < 0.1; ** indicates *p* < 0.05; and *** indicates *p* < 0.01. Inbred lines are as shown in Figure 3.

Variables	*PSY1*	*CYP97C*	*HYD3*	*CCD1*	Total Carotenoids	α-Carotene	β-Carotene	α-Cryptoxanthin	β-Cryptoxanthin	Lutein	Zeaxanthin
*PSY1*	-	0.49 **	0.70 ***	−0.29	0.46 **	0.13	0.12	0.07	0.41 **	0.23	0.52 ***
*CYP97C*	0.49 **	-	0.34 *	0.09	0.25	0.30	0.24	0.14	−0.06	0.26	0.03
*HYD3*	0.70 ***	0.34 *	-	0.10	0.25	−0.16	−0.05	−0.25	0.72 ***	−0.22	0.57 ***
*CCD1*	−0.29	0.09	0.10	-	−0.50 **	−0.37 *	−0.42 **	−0.52 ***	0.00	−0.42 **	−0.36 *
Total Carotenoids	0.46 **	0.25	0.25	−0.50 **	-	0.67 ***	0.79 ***	0.79 ***	0.45 **	0.76 ***	0.63 ***
α-Carotene	0.13	0.30	−0.16	−0.37 *	0.67 ***	-	0.90 ***	0.81 ***	0.05	0.66 ***	0.07
β-Carotene	0.12	0.24	−0.05	−0.42 **	0.79 ***	0.90 ***	-	0.85 ***	0.23	0.63 ***	0.26
α-Cryptoxanthin	0.07	0.14	−0.25	−0.52 ***	0.79 ***	0.81 ***	0.85 ***	-	0.11	0.77 ***	0.23
β-Cryptoxanthin	0.41 **	−0.06	0.72 ***	0.00	0.45 **	0.05	0.23	0.11	-	−0.08	0.61 ***
Lutein	0.23	0.26	−0.22	−0.42 **	0.76 ***	0.66 ***	0.63 ***	0.77 ***	−0.08	-	0.07
Zeaxanthin	0.52 ***	0.03	0.57 ***	−0.36 *	0.63 ***	0.07	0.26	0.23	0.61 ***	0.07	-

**Figure 5:**
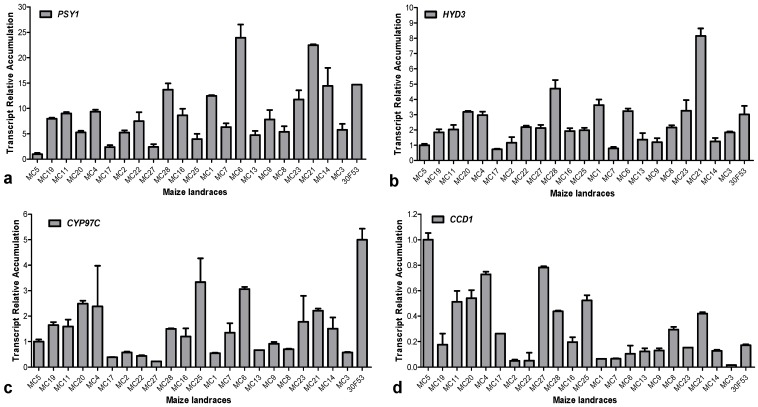
Expression profiles of carotenoid-related genes in grains from the maize varieties. (**a**) *PSY1*; (**b**) *HYD3*; (**c**) *CYP97C*; and (**d**) *CCD1*. The means of three replicates ± SD are shown. The white grain variety (MC5) was used as a reference sample for gene expression analysis calculated according to the 2^−ΔΔ*C*T^ method.

There was a remarkable difference in terms of both content and composition of carotenoids between the maize landraces; therefore, we investigated the expression levels of genes involved in the synthesis and degradation of carotenoids in these varieties to provide insight into the regulation of carotenogenesis in maize grains. The first step in determining provitamin A potential is to maximize biosynthetic flux and, therefore, total carotenoid synthesis. PSY catalyzes the first committed biosynthetic step and this enzyme has proven to be a major determinant of carotenoid content in several plants. In maize, three genes are currently known to encode the PSY enzyme (*PSY1*, *PSY2* and *PSY3*) [7,29]; however, *PSY1* is the only member of the PSY family whose transcript levels increased significantly during endosperm carotenogenesis and showed the expected correlation between transcripts and carotenoid content in the endosperm. Therefore, only *PSY1* was evaluated in the present study. Herein, the expression of *PSY1* varied substantially among landrace varieties, reaching values with a 24-fold higher expression compared to that of the white grain endosperm (MC5), which was used as reference sample (Figure 5). *PSY1* was the only evaluated gene whose expression in grains at 22 DAP was positively correlated (46%, *p* < 0.05) with the accumulation of carotenoids in grains at 48 DAP, corroborating its effect on the overall content of carotenoids.

The regulation of carotenogenesis has also been studied in other cultures, including tomato [33] and apple [34]. These studies have indicated that transcriptional regulation of the *PSY* gene appears to be the major regulatory mechanism responsible for determining the carotenoid pools in chromoplasts. Carotenoid biosynthesis also depends upon the availability of isoprenoid substrates, and there is substantial evidence indicating the existence of a metabolic feedback mechanism that modulates the supply of isoprenoid substrates and the accumulation of carotenoids and ABA [35]. Although *PSY1* expression was indeed correlated with carotenoid content in the present study, recent findings have shown that *PSY1* is polymorphic among maize varieties [36] and exhibits alternative splicing [37], which could, at least in part, explain the divergent expression patterns observed among the maize varieties. Post-transcriptional regulation has also been shown to control the enzymatic activity of PSY and PDS in chromoplasts [22]. Moreover, it is important to consider that other carotenogenic enzymes, upstream or downstream the conversion of carotene to phytoene, that were not evaluated in the present study, may also contribute to the overall production of carotenoids, as suggested by Vallabhaneni and Wurtzel [17].

### 3.3. Expression of Hydroxylases and Their Effects on Carotenoid Content

Although PSY play a role in the overall carotenoid content, other rate-limiting enzymatic steps might also significantly impact carotenoid composition. β-Carotene has the greatest provitamin A potential, and therefore, breeding to increase β-carotene accumulation requires enhanced flux to the β,β-branch of the pathway. It has previously been demonstrated that the use of breeding markers based on the *LCYε* gene is effective in selecting maize varieties with higher pathway flux toward the β,β-branch [16]. Therefore, the next challenge in breeding the varieties with high β-carotene levels in maize endosperm involves limiting β-carotene hydroxylation, as this process depletes the provitamin A pool by converting provitamin A compounds into xanthophylls that no longer have provitamin A activity [38].

As described above, the hydroxylation of the β-rings in α-carotene and β-carotene is potentially mediated by either HYD and CYP97A, while the hydroxylation of the ε-ring of α-carotene is performed by CYP97C (Figure 1). In maize, six functional genes encoding HYD have been identified [2], and carotenogenesis in the endosperm has been associated predominantly with *HYD3*. In the present study, the expression of *CYP97C* and *HYD3* varied substantially among landrace varieties, reaching values indicating five and eight-fold changes, respectively, compared to the white variety (Figure 5). When correlation analyses were performed regarding the expression of genes from the landrace varieties significant positive correlations were observed: *PSY1* and *HYD3* showed a 70% correlation (*p* < 0.01), *PSY1* and *CYP97C* showed a 49% correlation (*p* < 0.05), and *CYP97C* and *HYD3* showed a 34% (*p* < 0.1) correlation with one another. These correlations were similar among samples from multiple maize grain development stages, as presented above.

Quinlan *et al.* [38] expressed rice (*Oryza sativa*) CYP97A4 and CYP97C2 and maize HYD4 in *E. coli* engineered to accumulate both α-carotene (β,ε-rings) and β-carotene (β,β-rings) and showed that CYP97A preferred α-carotene over β-carotene as substrate and that the most efficient hydroxylation of β-carotene to zeaxanthin was achieved by HYD. Additionally, when HYD catalyzes the formation of zeinoxanthin (the precursor of lutein) from α-carotene in plants, this zeinoxanthin represents a pathway dead-end in terms of further conversion to lutein because the hydroxylation of the ε-ring by CYP97C is dependent on the interaction with CYP97A. It remains unclear, however, whether this preference also occurs *in planta*. Using a maize diversity core collection produced by “metabolite sorting”, it has previously been determined that *HYD3* transcript levels negatively correlate with high β-carotene levels and positively correlate with zeaxanthin levels [2]. In the present study, *PSY1* and *HYD3* expression in grains at 22 DAP was significantly correlated with the β-cryptoxanthin (41% and 72%, respectively) and zeaxanthin (52% and 57%, respectively) contents in grains at 48 DAP. For example, the variety MC21 exhibited the highest expression of *HYD3*, which resulted in high levels of β-cryptoxanthin and zeaxanthin, while the hybrid variety had the highest expression level of *CYP97C*, as well as high levels of α- and β-carotene and α-cryptoxanthin compared with the landraces. Therefore, two distinct correlation groups were observed: the α-carotene, β-carotene, α-cryptoxanthin and lutein levels correlated with one another, while the β-cryptoxanthin, zeaxanthin and *HYD3* expression levels were correlated. These results and the previous results obtained in *E. coli* assays [38] contribute to the understanding of the role of *HYD3* and *CYP97C* in the conversion of β-carotene to zeaxanthin or lutein in maize grains. However, other enzymes from each pathway branches should be investigated in order to obtain conclusive results.

**Figure 6:**
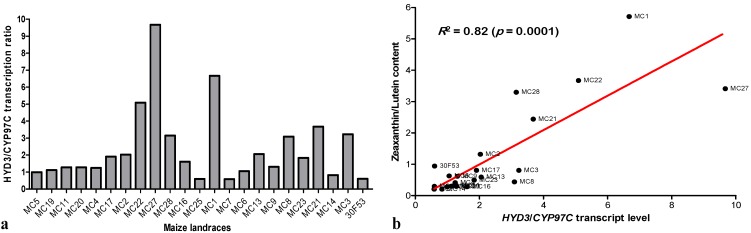
*HYD3*/*CYP97C* transcript ratio (**a**), and Pearson correlation (*R*) and statistical significance (*p*) for the comparison of the *HYD3*/*CYP97C* transcript ratio to the zeaxanthin/lutein content ratio (**b**). The gene expression levels of *HYD3* and *CYP97C* were determined in grains at 22 days after pollination (DAP), and the carotenoid content was determined in grains at 48 DAP. The maize inbred lines used are as indicated in Figure 3.

Although several efforts have been made to increase the levels of β-carotene in maize grains [2,16,19,21], there also exists much potential value in developing high zeaxanthin and high lutein maize varieties, as both of these compounds play important roles in ameliorating the progression of age-related macular degeneration, which is the leading cause of blindness in developed countries [24]. As such, in an attempt to predict the zeaxanthin/lutein ratio content in several maize landrace varieties, we calculated the *HYD3*/*CYP97C* expression ratios (Figure 6a). This approach appeared to be effective, as we observed a significant positive correlation (82%, *p* < 0.01) between the *HYD3*/*CYP97C* and zeaxanthin/lutein ratios (Figure 6b). Therefore, varieties with high *HYD3*/*CYP97C* expression ratios could be selected when the purpose of the breeding program is to enhance the zeaxanthin content in the grain, while varieties with low *HYD3*/*CYP97C* expression ratios could be selected when the objective is to improve lutein content.

Most of the cultivars evaluated showed higher expression of *HYD3*, which may be a consequence of the fact that HYD is able to form homodimers and occasionally acts in the β,ε branch of the carotenoid metabolic pathway [38]. In fact, most of the varieties whose *HYD3*/*CYP97C* ratio was approximately one had higher levels of lutein than zeaxanthin. The varieties whose *HYD3*/*CYP97C* ratio was approximately two had similar levels of these two carotenoids, while the varieties with the highest *HYD3*/*CYP97C* ratios had remarkably high levels of zeaxanthin (Figure 4 and Figure 6a, respectively). In this context, the evaluated germplasm contains promising varieties for biofortification efforts focused on increasing the levels of carotenoids synthesized in both branches of the biosynthetic pathway. Varieties MC27 and MC1, for example, had *HYD3*/*CYP97C* ratios of approximately 10 and 7, respectively, while the maize hybrid 30F53 had a ratio of less than 1 (Figure 6a). As such, these varieties are promising candidates for increasing zeaxanthin. In contrast, the increased expression of *HYD3* indicates a reduction in the maintenance of β-carotene in the cell (the levels of β-carotene 30F53 were higher than those in MC27 and MC1, for example). Therefore, varieties with low *HYD3*/*CYP97C* ratios—which are the case for several varieties, such as MC14 and MC7—may be useful in biofortification efforts aimed at enhancing the accumulation of provitamin A (Figure 6a). Furthermore, MC21 has a high total carotenoid content and a high *HYD3*/*CYP97C* ratio because of higher levels of β-cryptoxanthin and zeaxanthin relative to α-cryptoxanthin and lutein; therefore, it could be a promising target for down-regulating the expression of *HYD3* to reduce the levels of β-cryptoxanthin and zeaxanthin to levels that still provide a source for ABA (abscisic acid) biosynthesis (and thus not compromising seed development and defense) and achieve higher levels of β-carotene in the grain.

### 3.4. Expression Pattern of *ZmCCD1* in Maize Landraces and Its Correlation with Carotenoid Content

Carotenoid degradation is an important factor in determining total carotenoid accumulation and composition; however, it is largely unknown how the transcript levels of carotenoid cleavage-related genes correlate with carotenoid content and composition in mature kernels of maize varieties. Therefore, in addition to evaluating carotenogenic genes, we compared the carotenoid content with the expression levels of *ZmCCD1* in the maize germoplasm collection, for the purpose of investigating the role of the CCD1 enzyme in reducing carotenoid availability in maize grains.

The expression of *ZmCCD1* varied largely among the maize landraces (Figure 5) and, as expected, the white variety showed the highest expression levels. Interestingly, *ZmCCD1* expression was negatively correlated with the total carotenoid contents (50%), as well as with α-carotene (37%), β-carotene (42%), α-cryptoxanthin (52%), lutein (42%) and zeaxanthin (36%) levels (Table 2). Sun *et al.* [9] found that *ZmCCD1* cloned into *E. coli* effectively cleaved lycopene to produce pseudo-ionone, β-carotene to produce β-ionone, and zeaxanthin, to produce pseudo-ionone, β-ionone and 3-hydroxy-ionone, as indicated in Figure 1. Furthermore, *CCD1* expression levels were reported to be correlated with ripening, with a concomitant decrease in lutein content in strawberry [39]. Additionally, apocarotenoid volatiles have been identified from *E. coli* expressing *ZmCCD1* and producing ζ-carotene, lycopene, δ-carotene, β-carotene or zeaxanthin [40]. All of these data suggest that the CCD1 enzyme has a broad substrate specificity, and that the role of CCD1 in plants is not entirely understood. Furthermore, the *in planta* role of *ZmCCD1* may not necessarily be identical to the role observed in the artificial *E. coli* system, and carotenoids from the β,ε-branch (α-carotene, α-cryptoxanthin and lutein) may also be substrates of CCD1 in maize, as suggested by the negative correlations observed (Table 2).

Despite their important role in the cleavage of carotenoids, other CCDs that have not yet been characterized may also participate in this process and should thus be considered in future studies. Prediction algorithms (SignaIP 3.0) indicate that *Zm*CCD1 is devoid of a plastid targeting signal, suggesting that its site of action is the cytosol [9]. As such, we expect that other members of the CCD family are responsible for the cleavage of maize carotenoids in the plastid and that cleavage by CCD1 occurs after further transport of these intermediate apocarotenoids into the cytosol. In fact, it has been suggested that in *Medicago truncatula* roots, CCD7 may be the first cleavage enzyme involved in C13/C14 apocarotenoid biosynthesis induced by mycorrhizal fungal infection, acting in the plastid; then, following the export of the C27 intermediates, CCD1 would act in the cytosol [41].

## 4. Conclusions

As expected, significant quantitative and qualitative differences in the content and composition of carotenoids among the grains from different maize landraces were observed. Gene expression analyses showed that *PSY1*, *CYP97C* and *HYD3* were more highly expressed at the end of grain development and were positively correlated with the total carotenoid content. In contrast, *ZmCCD1* was more highly expressed at the beginning of grain development and was negatively correlated with the total carotenoid content, as well as with several individual carotenoids, suggesting that conversion to apocarotenoids is an important step in carotenoid accumulation, which must be investigated in biofortification efforts. The analysis of the expression of *HYD3* and *CYP97C* in maize landraces contributed to the understanding of the conversion of β-carotene to zeaxanthin or lutein, a step that limits the levels of provitamin A. The correlation between transcript profiles and carotenoid content in the germplasm collections studied here provides valuable knowledge and tools to use in biofortification efforts aimed at the improvement of provitamin A and non-provitamin A compounds in maize. Our knowledge of the carotenoid biosynthetic and catabolic pathways in maize, however, remains incomplete. Further investigations regarding allelic diversity, in addition to protein expression and enzyme activity, across diverse maize landraces may also help us to better understand and identify potential regulatory genes that may be relevant for improving the carotenoid content in maize grains using the natural genetic variation of this crop.
